# Segmentation of Polish Households Taking into Account Food Waste

**DOI:** 10.3390/foods9040379

**Published:** 2020-03-25

**Authors:** Beata Bilska, Marzena Tomaszewska, Danuta Kołożyn-Krajewska, Małgorzata Piecek

**Affiliations:** 1 Department of Food Gastronomy and Food Hygiene, Institute of Human Nutrition Sciences, Warsaw University of Life Sciences—SGGW, Nowoursynowska 159C St., 02−776 Warsaw, Poland; marzena_tomaszewska@sggw.pl (M.T.); danuta_kolozyn_krajewska@sggw.pl (D.K.-K.); 2 Polish Food Technologists’ Society, Nowoursynowska 166C St., 02−787 Warsaw, Poland; wrzosekmalgorzata@wp.pl

**Keywords:** food waste, food waste segmentation, households, consumers, cluster analysis, behavior, food practices

## Abstract

Currently, food waste is estimated at more than one-third of all food produced, and the primary responsibility for this phenomenon is attributed to households. Therefore, it seems reasonable to take action to limit food waste and to raise awareness about this link in the chain. To develop and implement educational programs addressed at consumers it is necessary to understand the factors determining food waste in households. Segmentation is a tool that can help effectively reach consumers who are to the greatest extent wasting food which identifies homogeneous clusters of consumers. The aim of this study was to perform segmentation to identify consumer groups with similar behaviors in relation to food, with particular emphasis on food wastage. We carried out segmentation on a representative sample of Polish people over 18 years of age and to identified three clusters of consumers. The three consumer segments diagnosed differed in sociodemographic terms, i.e., number of adults, number of children, subjective assessment of the financial situation, and percentage of spending on food. The segment exhibiting a high frequency of discarding food due to too large package size included single and double households.

## 1. Introduction

Waste, which is estimated at more than one-third of all food produced, is becoming a serious global problem that threatens the sustainable system, generating negative social, ecological, economic, and ethical consequences [1,2]. It is estimated that the level of food waste in the European Union, in 2012, was approximately 88 million tons [3]. With respect to the quantity of food produced, this value is approximately 20%. [3].

Food waste is produced along the various stages of the food supply chain [4]. The sectors contributing the most to food waste are households (47 million tons) and processing (17 million tons). These two sectors account for 72% of the EU’s food waste. Of the remaining 28% of food waste, 11 million tons (12%) comes from food service, nine million tons (11%) comes from primary production, and five million tons (5%) comes from wholesale and retail [3].

Despite the fact that there are multiple ways of decreasing the generation of waste, the common pillar is the prioritization of food waste prevention [5]. The second most attractive option involves distributing food surplus to groups affected by food poverty [6]. The need to ensure food security to people in food poverty has led to the development of new technologies in the food business that are aimed at preventing food losses and waste and at improving the quality of products recovered by charities [7,8]. A smart portable device associated with good prevention practices could potentially be useful to reduce food waste in the agrifood supply chain and in the food recovery field for charitable purposes [9].

The next option is converting food waste to animal feed [6]. According to Castrica et al. [10] feed product derived from food waste is compliant with the EU safety requirements, is nutritionally valuable, and has great potential.

It should be noted that within the structure of costs incurred in the food chain losses in the European Union, the highest level of wastage is generated by the last link, namely households (more than 50%, the cost of approximately 98 billion euros) [3]. Using data received from the EU-27, the BIO Intelligence, Paris, France, Service calculated that, at the household level, one household member discards approximately 76 kg of food per year. An analysis of food waste across the EU shows that there are substantial differences in the quantity per one household member. For example, the level recorded in the Czech Republic was 25 kg/person/year, whereas in the United Kingdom it was 133 kg per person per year. In Poland, the waste level was 54 kg of waste food per person per year [11]. Estimates show that approximately 60% of food discarded at this stage was still suitable for human consumption. Therefore, it seems reasonable to take action to limit the phenomenon of food waste and to raise awareness of the problem among consumers [3], especially because consumers are concerned about the scale of this phenomenon and believe that discarding food is wrong and evokes negative emotions (often shame and resentment) and ethical objections. It should be noted, however, that in the subjective assessment they do not see themselves as the main source of discarded food imputing responsibility to other links in the food chain [12]. In addition to knowledge and awareness, food waste is affected by many factors which cannot be interpreted in isolation. The tendency to discard is rather the result of how a household copes with all activities related to food. This process consists of activities such as planning and doing shopping, food storage, meal preparation, and consumption [3].

One of the main factors that motivates action to limit food waste more strongly than environmental and prosocial awareness is a desire to minimize one’s own expenses. In the context of loss, consumers often mention losing time for buying products, bringing them home, and preparing a meal that eventually is not consumed or is partially consumed. The link between ecological impacts such as global warming or excessive consumption of natural resources is not clearly identified with the quantity of food that is wasted [12]. The research by Neff et al. [13] and Qi and Roe [14] has shown that only 40% and 58.4% of the respondents are aware of the consequences and the negative influence of food waste on the environment, respectively. Therefore, the key is to build awareness and knowledge that discarding food unduly wastes personal financial resources at the same time exerting a negative impact on the environment [12].

Food waste is the result of many complex factors and consumer behavior [15,16]. Among the edible fraction, food is more likely to be thrown in the bin resulting from excess left on the plate after eating, excess prepared but not served, and products opened but not finished [17]. According to Bilska et al. [18], the most common causes of food waste include food being spoiled and missing the expiry date. Zielińska et al. [19] noticed that the majority of respondents have difficulty distinguishing and properly understanding the terms “use by” and “best before” on the label. As pointed by Ramírez et al. [20], an electronic nose could be useful for consumers, as well as meat industries and related food services, that would allow real-time characterization of these products. The results have shown that pork meat trays should not be labelled with the same expiration date, because of observed wide variation in the typical hygiene and sanitary quality parameters [20].

According to many researchers [16,21,22,23], the quantity of wasted food is correlated with demographic factors, notably household size. Single households waste more per person, which implies an economic scale associated with the size of commercially available packages. The Waste and Resources Action Programme Report [15] indicated that young people, both single, young families, young people, and children, tend to waste more food. Similarly, Hanssen et al. [24] pointed out that young people tend to waste more food, which is why educational programs are addressed largely to these groups. According to Aschemann-Witzel et al. [25], demographic data does not play as important a role in explaining food waste at the consumer level as do psychographics. The Fusions Report draws attention to three factors, namely social factors (type of household and family stage), individual behavior, as well as lack of awareness, knowledge, and skills [26].

A good understanding of the factors determining food waste in a household [27] facilitates the development and implementation of consumer education programs, A tool that can effectively help reach consumers who waste the most food is segmentation, which is considered one of the most accepted and important concepts, both in academic research and real world practice [28]. A one-size-fits-all approach cannot meet all the varied requirements; therefore, companies need to consider differentiated approaches to customers, products, and supply [29]. As pointed by French [30], segmentation adds real value to most social programs and is a common technique that has been applied as part of social marketing programs.

According to Johnson et al. [31], market segmentation is a valuable tool for identifying consumers with similar needs who are likely to respond in a similar way to marketing communication. Each segment requires individual products or marketing mixes [32,33]. These rules also apply to educational campaigns.

Demographic and socioeconomic variables are the most popular basic elements for segmentation, because the information is relatively easy to collect and measure and is the best example of a priori descriptive methods. These variables include age, sex, size of household, household income, profession, and education [31].

The aim of the study was to perform segmentation to identify consumer groups with similar behavior in relation to food, with particular emphasis on its wastage. The question was the following: (1) Whether food waste in households is associated with sociodemographic features such as household size, personal composition, subjective assessment of the financial situation, and level of spending on food as well as (2) the impact these factors have on food waste.

## 2. Materials and Methods

### 2.1. Sample Collection

The survey was conducted in February and March 2019, among a group of 1115 adult consumers using computer-assisted personal interviews (CAPIs). The selection of the sampling from the address survey of the Central Statistical Office in Poland, Warsaw fulfilled the condition of representativeness of the general population for the Polish people over 18 years in terms of gender, age, and size of the place of residence. The survey was conducted in each of the sixteen voivodeships in Poland. After drawing the starting addresses, the so-called method of random route was used in the selection of the sample.

The factors taken into account in the segmentation of the respondents included characteristics such as the number of adults in the household (over 18 years), number of children, subjective assessment of the financial situation of the household, and percentage of spending on food. The detailed sociodemographic profile of the respondents is presented in Table 1. The surveyed households were dominated by families consisting of two adults. Much lower percentage values concerned households which consisted of one (and three or more adults). The majority of the respondents declared that there were no minors in their households. As for families with children (under 18 years), the survey was dominated by families with one child (respondents were asked about their financial situation). For this purpose, were used subjective financial assessments as a measure of financial well-being. A majority of the respondents declared an average financial situation. The actual survey was preceded by a pilot study conducted on 30 people.

### 2.2. Questionnaire

**Figure 1:**
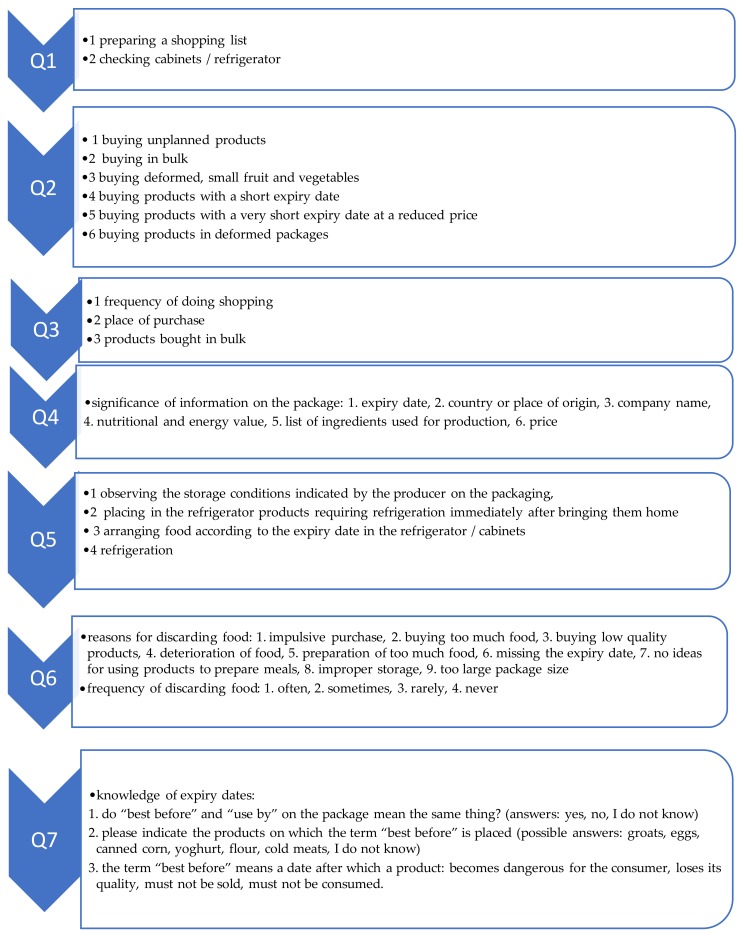
Schematic diagram of the issues used for segmentation of households.

The study was performed using a specifically designed questionnaire which consisted of two parts. The first part contained questions regarding consumer behavior and knowledge in relation to food was divided into seven areas (Figure 1).

These topics were selected on the basis of the literature [34,35,36], which showed that these aspects affect food waste in households. The first group of questions concerned preparation for shopping and behavior during shopping (using a 5-point scale “always, usually, sometimes, rarely, never”) (Figure 1Q1,Q2). Food products were divided into 32 groups, and then the respondents were asked about the place of purchase (market, small private shop, small chain store, discount store, hypermarket, specialized shop, and via the internet) and frequency of doing shopping (daily, on average every other day, on average 1−2 times a week, on average 1−2 times per month, less than once a month, never) (Figure 1Q3). In addition, the respondents were asked to identify food products purchased in bulk (possibility of indicating several responses) (Figure 1Q3). The survey was also aimed at examining the degree to which information on the product package was important to the respondents (using a 5-point scale from “strongly agree” to “strongly disagree”) (Figure 1Q4). A significant issue is the proper storage of food at home (Figure 1Q5). This question used a 5-point scale ranging from “always” to “never”. In addition, the respondents were asked to select 20 products kept in the refrigerator from among the products listed (Figure 1Q5). The key issue was 32 food groups waste, i.e., the reasons and frequency (Figure 1Q6). In the questions concerning the reasons for discarding food, the respondents could indicate several responses. The respondents were also asked questions regarding the minimum “best before” date (Figure 1Q7). The scale was deliberately differentiated and adapted to the type of question.

The second section contained questions concerning the demographic and social affiliation of the respondents as follows: number of people in household, number of children in household, subjective assessment of the financial situation, and portion of the household budget spent on food.

### 2.3. Statistical Analysis

A taxonomic analysis was performed using Statistica 12.1. PL (StatSoft, Cracow, Poland). Its aim was to select several groups of respondents differing in behavior related to food waste. A multidimensional cluster analysis was used for this purpose. A non-hierarchical k-means method was used to create clusters because it allows establishment of a priori, of the number of clusters. This algorithm uses the Euclidean distance. Homogeneous clusters of respondents were created based on the average frequency rate. The starting point of the analysis was the identification of a specified number of segments of respondents on the basis of their descriptive (“sociodemographic”) characteristics. In this way, 35 different segments were created as follows: number of adults in the household, number of children, subjective assessment of the financial situation, and percentage of spending on food. Subsequently, based on the average frequency rate (for each segment) illustrating discarding 32 groups of non-consumed food products, the respondent segments (35) were grouped into three uniform clusters.

Cluster analysis was complemented by the examination of the significance of differences between the mean levels for each element (consisting of a multidimensional criterion for the creation of clusters) in the identified clusters. The null hypothesis of equality of the mean value (calculated for each cluster) was verified using the Fisher–Snedecor F test, and a post-hoc analysis was performed using the LSD (least significant difference). test. This allowed identification of homogeneous groups of arithmetic means. This verification was carried out with a significance level of *p* = 0.05. The basis of inferences was the test probability values “*p*”. This enabled indication of homogeneous arithmetic mean groups. The null hypothesis was rejected when *p* < 0.05.

A preliminary analysis was performed assuming 2, 3, 4, and 5 expected clusters. This analysis showed that the clearest profile of the groupings of elements (segments) in the individual clusters was obtained when assuming 3 clusters.

## 3. Results

### 3.1. Sociodemographic Profile of Segments

Segmentation was carried out on a representative sample of Polish people over 18 years of age and we identified three clusters with varying percentage share as follows: Cluster 1 “saving food” 41.8%, Cluster 2 “wasting vegetables and fruit” 46.3%, and Cluster 3 “wasting food” 11.9%. The three clusters differ significantly in sociodemographic terms (Table 2). More than half of households in Cluster 1 are formed by two adults and one in five households consists of a single person, or three or more adults. In Cluster 2, there are no single households and it is dominated by families consisting of two adults with one child. Segment 3 is dominated by single households. All three segments are dominated by households without children, which definitively prevail in Cluster 3. Nearly half of households forming Cluster 1 declared a good financial situation and a small proportion of spending on food. Most households classified in Segments 2 and 3 are characterized by average financial situation. In Cluster 3 almost half of the people declared a large proportion of spending on food.

### 3.2. Characteristics of the Defined Segments Taking into Account Common Features

The analysis of variance (F test) showed that the three segments diagnosed were similar in terms of preparation for shopping. Almost a quarter of the consumers declared that the did not check their supplies before going shopping (22.6%) or prepare a shopping list (27%) (answers “never” and “rarely”). It was also found that the people classified in the three segments were, to the same extent, willing to purchase unplanned products (18.9% response “always” or “usually”). A relatively small percentage of the respondents declared that they “always” or “usually” buy food in bulk (16.6%).

It was also found that the people classified in the three segments generally did not arrange food based on the expiry date in the fridge and cupboard (41.8% of answers “rarely” or “never”).

The analysis of variance (F-test) showed that in the case of products such as bread, cereals, and legumes, the frequency of discarding was similar for all three clusters. Particularly noteworthy is that bread was one of the products most commonly discarded by the Polish respondents. Almost a quarter (23.8%) declared that they “often” and “sometimes” discard this product. There was also no difference among the three segments in terms of the frequency of buying 23 groups of food products, including bread, which was purchased by the vast majority (82.3%) of the respondents daily or on average every other day.

Generally, there were no differences in the reasons for discarding food, with the exception of package size. The vast majority of the respondents declared that they discarded food because of its deterioration (65.2%) and missed expiry date (42%). Nearly one-quarter of the respondents wasted food because they prepared (26.5%) or bought (22.2%) too much.

The diagnosed clusters similarly interpreted the meaning of the “best before” and “use by” dates. Almost half of the respondents (42.8%) believed that these dates mean the same thing, more than one third of the respondents (36%) were of the opposite view, and every fifth respondent (21.2%) answered “I do not know”. The respondents in the three segments failed to correctly indicate the products which had a “best before” date. Forty per cent of the respondents correctly indicated groats, and nearly 2% more (41.7%) incorrectly indicated yoghurt and buttermilk. A similar percentage of the respondents believed that the term “best before” meant the date after which a product becomes dangerous for the consumer (39.8%) or loses its quality (37.4%).

### 3.3. Description of the Defined Segments in Terms of Handling Food

The “saving food” cluster, more rarely than the other segments, declare buying fresh red meat (Table 3).

People in Cluster 1 least frequently refrigerate fresh herbs and carrots as compared with the other clusters. This segment stood out with a lower share of persons declaring discarding 13 groups of products, both of animal origin (e.g., milk and dairy products, eggs, fresh poultry meat, sausages) and of plant origin (e.g., potatoes, fruit and vegetable products) as compared with the other segments (Table 4). People in the “saving food” cluster do not discard any food product more often than the other two segments.

People in the “wasting vegetables and fruit” cluster most frequently refrigerate tomatoes as compared with the other clusters (Table 3). People in this cluster most frequently buy bread in small private shops, grain products and fresh red meat in supermarkets, and fresh fish in specialized shops (Table 4).

**Figure 2:**
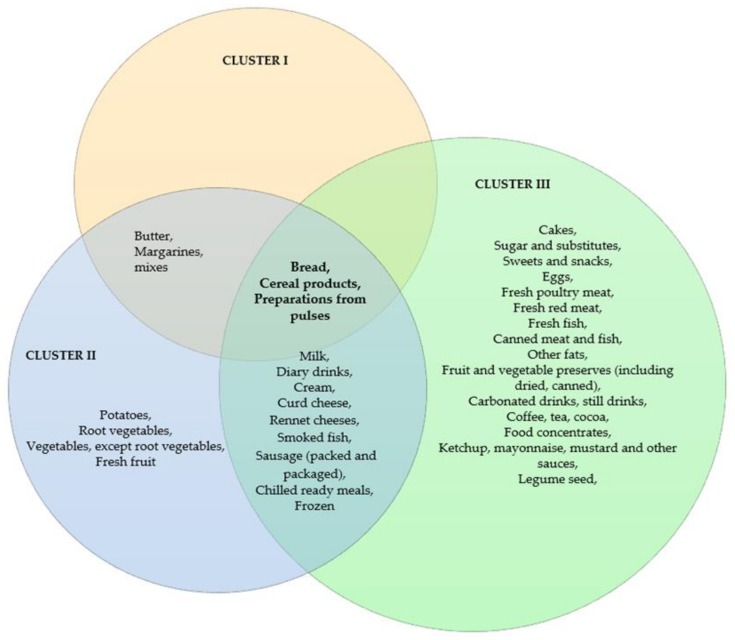
The highest frequency of discarding individual product groups declared by the three segments identified.

Cluster 2 was characterized by people who declare most frequently discarding fresh fruit, potatoes, root vegetables and other vegetables as compared with the other two clusters (Table 4, Figure 2). More people classified in this segment believe that the term “best before” means the date after which a product can be consumed (Table 3).

Households classified in the “wasting food” cluster purchase food products with a very short “best before” or with short “use by” dates which are offered at a discounted price more often than others. Nevertheless, for the people classified in Cluster 3 the expiry date and price are less important than for those in the other clusters. It should also be noted that the respondents forming Cluster 3 more often than others buy deformed, too little fruit and vegetables, and food products in deformed packaging (Table 3).

Cluster 3 includes respondents buying less milk and fresh fruit. They buy fresh fish, canned meat and fish, legume seeds and preserves, ready meals and frozen foods more often as compared with Clusters 1 and 2.

The factor differentiating the clusters is also the place of purchase. The LSD test indicates that people in Cluster 3 most frequently buy cakes, rennet cheese, fresh poultry meat, root vegetables, ketchup and other sauces, coffee, tea and cocoa in small chain stores, and sugar and substitutes-in markets as compared with the other clusters (Table 4).

People in Cluster 3 less frequently buy sugar and substitutes in discount stores, smoked fish in small private shops, and fresh fish in specialized shops as compared with the other clusters (Table 4).

The factor that could influence the level of food waste in households is the model of buying food products “in bulk”. It has been shown that buying in bulk 3 groups of products substantially distinguishes between the clusters in the test sample. Cluster 3 is distinguished by people who less frequently buy in bulk cereal products and fats (Table 3). In addition, the respondents belonging to this cluster more frequently do not observe the storage conditions specified by the manufacturer on the package, or place food products requiring refrigeration temperatures in the refrigerator immediately after bringing them home as compared with the other clusters. Cluster 3 has less people keeping in the refrigerator fresh poultry meat, fresh fish, yogurt, buttermilk, fresh milk and butter. In contrast, a higher percentage of the respondents refrigerate onion (Table 4).

In the course of studies, it was also shown that people in Cluster 3 most frequently indicated too large package size as the main reason for discarding food as compared with the other clusters.

The “wasting food” cluster is characterized by the people who most frequently discard 15 groups of food products, including sweets and salty snacks, eggs, fresh poultry and red meat, fresh fish, canned meat and fish, and ready meals (Table 4, Figure 2).

People in Cluster 3 least frequently indicated that they do not know which products could contain the term “best before” as compared with the other clusters (Table 3).

**Figure 3:**
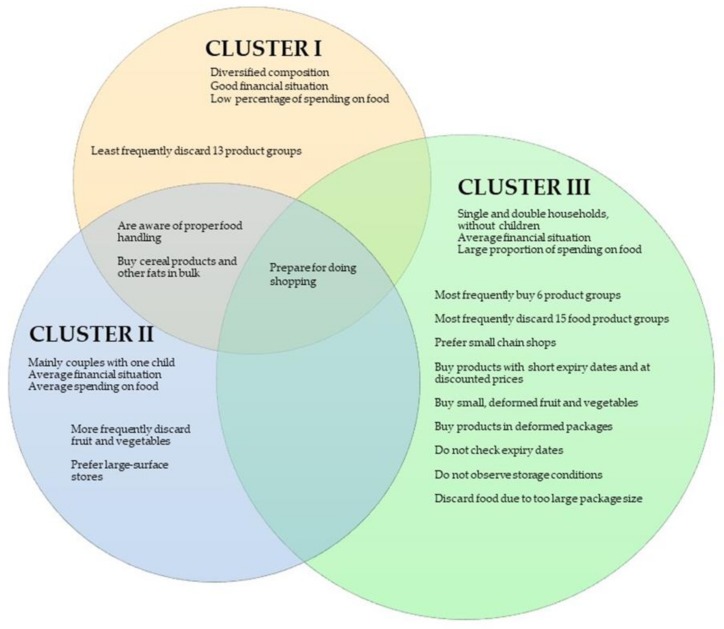
Characteristics differentiating three segments diagnosed.

The features differentiating the segments are shown in Figure 3.

## 4. Discussion

This study diagnosed three consumer segments differing in sociodemographic terms, i.e., number of adults, number of children, subjective assessment of the financial situation, and percentage of spending on food. Despite the many differences observed in the behavior of the three segments, several similarities were also found. The respondents in the three clusters generally do not check their product stocks at home before going shopping, or prepare shopping lists, and “sometimes, often” buy unplanned items. According to many researchers [16,21,23,37] careful planning of grocery shopping is an effective tool for the prevention of food waste. While conscious estimation of demand for a given product and its corresponding quantity relevant to the number of members in the household can reduce buying excessive products, which consequently have no chance of being consumed [38]. It has been estimated that such preparation for shopping could reduce the quantity of food discarded by a household by approximately 20% per one family member [12].

The respondents from all three segments do not tend to arrange products by the expiry date in their cupboards or refrigerator. Sorting products from the shortest to the longest expiry dates undoubtedly helps in the proper management of food storage in the household [10,39]. Consumers can use practical advice as well as all sorts of ‘’goodies’’ to ensure the proper storage of food at home. A good example of a campaign directed to consumers is that conducted by the Netherlands Nutrition Centre, The Hague, the Netherlands which is in an accessible way helps not only to gain knowledge of the proper handling of food at home, but also to acquire practical accessories useful in food management [40].

All three segments discarded bread with almost the same high frequency. Many researchers have observed that the product is among the most frequently wasted foods [21,25,41]. According to [42], consumers require that bread meets high requirements for freshness, and stale bread is most often thrown away by them. A survey of 1000 Austrians aged 15 and over showed that 78% of respondents rated freshness as the most important attribute of bread [43].

In addition, the respondents belonging to the three segments have difficulty interpreting the “use by” or “best before” dates placed on packages. Boxstael et al. [44] also showed inadequate consumer knowledge of these two terms. According to many researchers [13,15,22,45,46,47,48], various different languages on date labels and misunderstanding of the meaning of expiry dates is closely associated with a higher frequency of discarding food. As assessed in the UK, approximately one fifth of avoidable food waste is wasted due to the lack of understanding, by 40% to 49% of consumers, of the information concerning the ‘’best before’’ dates on the packaging. Therefore, it is also necessary to educate consumers, in terms of distinguishing between the “use by” and “best before” dates on food packaging. Thanks to the campaign ‘’Love Food Hate Waste’’ conducted in the UK in 2008, avoidable food waste was reduced in households by 3% [11].

The most distinctive segment were single and double households without children (Segment 3), exhibiting the highest frequency of discarding 15 food product groups. Mallinson et al. [49] made a similar observation that high wastage of food occurs in the segment consisting of single households. Many available studies indicate a correlation between the quantity of wasted food and household size. Single households waste much food per person [21,22,50,51,52].

Cluster 2 consisting primarily of families with children is characterized by the highest frequency of discarding fruit and vegetables. The available research indicates that the number of children can impinge disproportionately on the level of food waste due to the unpredictability of behavior and food preferences of children, and parents desire to serve fresh and the highest quality products [53,54,55].

Most people in Segment 1 declared a good financial situation, the lowest level of spending on food, as well as the lowest frequency of discarding 13 product groups. This observation is confirmed by the study by Mallinson et al. [49], conducted in the UK, which showed that the cluster with the lowest percentage of consumers discarding food were people with the highest income levels as compared with the other clusters. Pearson et al. [56] arrived at an opposite conclusion finding that higher income is associated with higher food waste, which is relatively cheaper than other goods.

The reason for discarding food by small households, which was observed in our study in Poland, was too large package size. This observation was confirmed in studies [21,52] which showed that it is more profitable for small households to purchase goods in larger packages. According to Williams et al. [57], approximately 20% of food is wasted because of a package which is too large or difficult to empty. Products in smaller packages are disproportionately expensive, so despite being aware of the inability to use a product, consumers buy larger packages. A factor stronger than the awareness of wastage which determines consumers decisions is a significant difference in price based on product weight. These decisions result in an increase in food waste in households in total by approximately 20% to 25% [58].

The quantity of food wasted in households is determined by the frequency and place of purchasing. The publication by Williams et al. [57] and Glanz [58] indicated that most food is discarded when consumers shop exclusively in large supermarkets. This quantity decreases when food products are purchased in a variety of commercial facilities, including in small shops and local markets. In the case of self-supply, the quantity of wasted food is the lowest because people are more aware of the amount of work, time and effort related to its production, breeding and growing. More frequent shopping also minimizes the quantity of discarded products because the planning perspective and current demand for food are not far away [12]. These observations have not been confirmed in our study. The “wasting food” segment prefers small chain shops. In addition, the segment buys and discards fresh and canned fish more often than the other two segments.

The respondents assigned to the “wasting food” cluster most frequently buy products with short expiry dates and at a discounted price, while noting the importance of low prices when buying food. As has been demonstrated by Koivupuro et al. [22] and Schmidt and Matthies [59], the factor exerting excessive influence on purchasing are marketing activities (discounts) that stimulate impulsive purchases. Nevertheless, researchers have shown that families who often buy food at a reduced price or consider low prices as an important factor when shopping, waste less food. Studies show, on the one hand, that households in which the price of food is an important determinant of purchase are more willing to use promotional offers, including “buy 2 and get 1 free”, and purchase products at a discount, waste less food [22,59]. On the other hand, households that spend more money on groceries per person tend to generate more food waste [12]. The factor motivating some consumer groups to buy food with short expiry dates is an eco-friendly attitude and counteracting food waste rather than a price reduction ensuring only a financial advantage. Increasing consumer awareness of the problem of food waste has a positive effect on a greater desire to prevent this phenomenon, and sometimes it is a stronger incentive than the economic aspect [59]. A factor that can prevent food waste at the stage of selecting food products by consumers in retail facilities is the willingness to buy “imperfect” food, i.e., undersized, with uneven staining, slightly distorted, in torn bulk package, including short expiry dates [12]. It should be noted that people in the “wasting food” segment are more likely than other clusters to buy deformed food in crooked packaging, as well as with short expiry dates, but their motivation is unknown. This segment more often than other segments buys ready frozen and chilled meals, which are also often discarded. According to Mallinson et al. [49], the use of convenience food restricts the purchase of products and semi-finished products required for conventional cooking, and therefore it can be deduced that it has a positive effect on the level of food waste in households. In the Swedish study by [60] it was demonstrated that such a correlation is not present. The respondents, in Segment 3, that wasted the most food are characterized by improper food storage and disregard for storage conditions specified by the manufacturer on the packaging. The fact that the persons belonging to this cluster less frequently keep unstable products under refrigeration is disturbing. According to Terpstra et al. [61], consumers have some knowledge of food storage, but do not always use it, for example, improperly store vegetables and set too high temperatures in the refrigerator. While Visschers et al. [62] found no direct relationship between knowledge about storage and the quantity of wasted food. According to Papargyropoulou et al. [6], households that improperly store food contribute to its waste.

Many studies have pointed to the insufficient knowledge of consumers concerning issues related to temperature during the technological process [63,64,65,66]. Meanwhile, temperature control during the manufacturing process is one of the primary tools in controlling the growth of microorganisms in food. Failure to observe the recommended values is the main cause of the proliferation of microbial cells and, consequently, a number of risks, including food poisoning [67]. According to Ishangulyyev et al. [68], consumers should be educated, among others, in financial management, interpretation of expiry dates on packaging, and proper storage of food.

### Limitations and Further Research

One limitation of this study is that food waste behavior included self-reported measures by respondents, which could be biased. Future studies should consider other sociodemographic factors not included in this segmentation, i.e., gender, age and other factors related to lifestyle, such as meals in the restaurant and ordering meals home. In our study, the segmentation used seven groups of issues and, in the future, studies should be extended to include other factors related to food preparation, planning portions, and use of leftovers.

It is also necessary to further analyze the issues raised in the study to learn the motives that guide persons purchasing deformed fruit and vegetables, and food products in deformed packages. To do this, qualitative research could be used.

In our study, the respondents answered to the question “how often do you discard food products?”, but other study methods should still be used to specify the quantity of wasted food.

## 5. Conclusions

Three segments differing in size, composition, assessment of the economic situation, and the level of spending on food were identified.

The segment dominated by single and double households without children exhibited a high frequency of discarding food due to too large package. At the same time, this segment did not store food in accordance with the manufacturer’s instructions, which could also contribute to its waste.

Segment 2 formed mostly by couples with one child and preferring large-surface stores was distinguished by the highest frequency of discarding fruit and vegetables.

People in Segment 1 declared the lowest frequency of discarding 13 product groups due to a good financial situation and the lowest level of spending on food.

The identified segments were similar in terms of the way of preparing for shopping and knowledge of the term “best before”.

Knowledge of the factors influencing consumer behavior related to food waste is essential for the development and implementation of effective education programs in order to reduce this negative phenomenon. It should be borne in mind that consumers are a heterogeneous population and it is necessary to divide them into clusters. Our study showed that segmentation is an effective tool to identify consumers with similar sociodemographic characteristics and behaving similarly in terms of purchase, storage, as well as the causes and frequency of discarding food. Such separate segments require the development of educational programs that most effectively respond to their needs and bring the desired effect which is to reduce food waste.

## Figures and Tables

**Table 1:** Sociodemographic profile of consumer groups (*n* = 1115).

Feature	Characteristics	%
No. of people over 18 years in household	1 2 3 or more	17.2 61.2 21.6
No. of children in household	0 1 2 or more	72.7 17.3 10.0
Subjective assessment of the financial situation	Good Average Refusal to provide an answer	41.6 57.6 0.8
Portion of the household budget for food expenditure	Large (100%–61%) Average (60%–40%) Small (39%–0%) Hard to say	12.3 50.2 27.7 9.8

**Table 2:** Sociodemographic profile of clusters.

Feature	% Share in the Segment	-		
		Saving Food	Wasting Vegetables and Fruit	Wasting Food
**Number of people**	1 adult	22.4	0.0	**68.2**
	2 adults	58.2	**72.6**	31.7
	3 and more adults	19.4	**27.4**	8.5
	At least 3 persons	37.4	**51.7**	15.5
**Number of children**	No children	73.4	73.2	**93.8**
	One child	16.0	**18.3**	0.0
	At least two children	**10.6**	8.5	6.2
**Financial situation**	Good *	**50.1**	33.6	33.3
	Average *	47.2	**64.8**	**66.7**
**Proportion of spending on food**	Low (0−39%) *	**33.6**	27.8	0.0
	Average (40−60%) *	47.9	**66.2**	53.5
	High (61−100%) *	12.0	4.4	**46.5**

* In the case of Segments 1 and 2, the numbers do not add up to 100% due to no response to a question.

**Table 3:** Selected aspects of food handling for the identified segments, average level of frequency rate, arithmetic mean and the results of the variance analysis and the LSD test.

-	Saving Food	Wasting Vegetables and Fruit	Wasting Food	p-Value	-	Saving Food	Wasting Vegetables and Fruit	Wasting Food	p-Value
-	Shopping Frequency (Arithmetic Mean **)			-	-	Refrigeration (Average Level of Frequency Rate *)			-
**Milk**	3.05 ^a^	3.09 ^a^	3.42 ^b^	0.038	Fresh poultry meat	0.80 ^ab^	0.86 ^b^	0.75 ^a^	0.042
**Fresh red meat**	3.79 ^b^	3.54 ^a^	3.47 ^a^	0.002	Fresh fish	0.76 ^ab^	0.83 ^b^	0.68 ^a^	0.010
**Fresh fish**	4.40 ^a^	4.34 ^a^	3.86 ^b^	0.001	Yogurt, buttermilk	0.91 ^ab^	0.94 ^b^	0.83 ^a^	0.031
**Canned meat and fish**	4.85 ^b^	4.69 ^b^	4.09 ^a^	0.000	Fresh milk	0.84 ^b^	0.89 ^b^	0.67 ^a^	0.000
**Fresh fruit**	3.02 ^a^	3.01 ^a^	3.45 ^b^	0.002	Carrot	0.21 ^a^	0.36 ^b^	0.32 ^b^	0.040
**Fruit and vegetable preserves (including dried, canned)**	4.48 ^b^	4.39 ^ab^	4.13 ^a^	0.010	Butter	0.84 ^b^	0.87 ^b^	0.75 ^a^	0.049
**Legume seeds**	4.69^b^	4.74 ^b^	4.11 ^a^	0.000	Tomatoes	0.32 ^a^	0.42 ^b^	0.34 ^a^	0.040
**Chilled ready meals**	4.72 ^b^	4.76 ^b^	4.35 ^a^	0.005	Onion	0.14 ^a^	0.17 ^a^	0.30 ^b^	0.006
**Frozen food**	4.57 ^b^	4.59 ^b^	4.27 ^a^	0.029	Juices after opening	0.63 ^ab^	0.71 ^b^	0.54 ^a^	0.035
-					Fresh herbs	0.05 ^a^	0.14 ^b^	0.11 ^b^	0.008
**Frequency of action** (**Arithmetic mean** **)				-	**Purchase frequency** (Arithmetic mean **)				-
**Observance of the storage conditions specified by the manufacturer**	2.17 ^a^	2.10 ^a^	2.45 ^b^	0.012	Deformed, small fruit and vegetables	4.17 ^b^	4.27 ^b^	3.71 ^a^	0.002
**Refrigeration of food immediately after bringing home**	1.67 ^a^	1.60 ^a^	2.05 ^b^	0.002	Food with short expiry dates	3.69 ^b^	3.74 ^b^	3.42 ^a^	0.005
**Significance of the information on the label** (Average value **)				-	Food with short expiry dates at a discounted price	3.73 ^b^	3.76 ^b^	3.39 ^a^	0.016
**Expiry date**	1.43 ^a^	1.35 ^a^	1.76 ^b^	0.001	Food products in a deformed package	4.09 ^b^	4.23 ^b^	3.85 ^a^	0.027
**Price**	1.56 ^a^	1.47 ^a^	1.83 ^b^	0.009	**Buying in bulk** (Average level of frequency rate *)				-
**Reason for discarding food** (Average **level of** frequency rate *)				-	Cereal products	0.56 ^b^	0.61 ^b^	0.37 ^a^	0.018
**Too big package size**	0.16 ^a^	0.15 ^a^	0.37 ^b^	0.001	Other fats	0.32 ^b^	0.33 ^b^	0.15 ^a^	0.041
**-**					Coffee, tea, cocoa	0.60 ^ab^	0.65 ^b^	0.47 ^a^	0.043
**The term “best before” means** (Average **level of** frequency rate *)				-	**Products on which the phrase “best before” is placed** (Average level of frequency rate *)				-
**Date after which the product can be consumed**	0.08 ^a^	0.14 ^b^	0.06 ^a^	0.048	Do not know	0.27 ^b^	0.22 ^b^	0.11 ^a^	0.015

An identical letter at the arithmetic mean value or frequency rate means that there are no significant differences between the clusters. * The highest frequency rate means the highest frequency. ** Rank order for reported behavior “1” being the highest reported frequency and “5” being the lowest reported frequency.

**Table 4:** Frequency of discarding 32 groups of food products and place of purchase, average level of frequency rate for the designated clusters, and the results of the variance analysis and the LSD test.

-	Frequency of Discarding Food Products			p-Value	Place of Shopping			p-Value	-
-	Average Level of Frequency Rate *				Average Level of Frequency Rate *				-
	Cluster 1	Cluster 2	Cluster 3		Cluster 1	Cluster 2	Cluster 3		Place of Shopping
**Bread**	0.58	0.69	0.60	ns	0.45 ^b^	0.54 ^c^	0.35 ^a^	0.007	Small private shop
**Cereal products**	0.42	0.45	0.49	ns	0.08 ^a^	0.16 ^b^	0.05 ^a^	0.045	Large-format shop
**Cakes**	0.42 ^a^	0.48 ^a^	0.60 ^b^	0.009	0.08 ^a^	0.05 ^a^	0.18 ^b^	0.041	Small chain store
**Sugar and substitutes**	0.21 ^a^	0.24 ^a^	0.40 ^b^	0.01	0.03 ^a^	0.02 ^a^	0.11 ^b^	0.009	Bazaar
					0.67 ^b^	0.62 ^b^	0.49 ^a^	0.021	Discount store
**Sweets and snacks**	0.20 ^a^	0.28 ^a^	0.49 ^b^	0.000	ns	ns	ns	ns	ns
**Milk**	0.38 ^a^	0.55 ^b^	0.54 ^b^	0.001	ns	ns	ns	ns	ns
**Diary drinks**	0.36 ^a^	0.59 ^b^	0.55 ^b^	0.000	ns	ns	ns	ns	ns
**Cream**	0.33 ^a^	0.55 ^b^	0.61 ^b^	0.000	ns	ns	ns	ns	ns
**Curd cheese**	0.34 ^a^	0.50 ^b^	0.47 ^b^	0.000	ns	ns	ns	ns	ns
**Rennet cheeses**	0.30 ^a^	0.46 ^b^	0.53 ^b^	0.004	0.04 ^a^	0.02 ^a^	0.17 ^b^	0.001	Small chain store
**Eggs**	0.18 ^a^	0.29 ^b^	0.44 ^c^	0.000	ns	ns	ns	ns	ns
**Fresh poultry meat**	0.19 ^a^	0.28 ^b^	0.39 ^c^	0.001	0.06 ^a^	0.04 ^a^	0.16 ^b^	0.005	Small chain store
**Fresh red meat**	0.21 ^a^	0.26 ^a^	0.39 ^b^	0.000	0.07 ^a^	0.15 ^b^	0.07 ^a^	0.023	Large-format shop
**Fresh fish**	0.20 ^a^	0.29 ^a^	0.48 ^b^	0.000	0.20 ^b^	0.34 ^c^	0.08 ^a^	0.001	Specialized store
**Sausage (packed and packaged)**	0.43 ^a^	0.60 ^b^	0.56 ^b^	0.000	ns	ns	ns	ns	ns
**Smoked fish**	0.26 ^a^	0.34 ^b^	0.43 ^b^	0.008	0.22 ^b^	0.22 ^b^	0.10 ^a^	0.041	Small private shop
					0.07 ^ab^	0.04 ^a^	0.12 ^b^	0.045	Small chain store
**Canned meat and fish**	0.24 ^a^	0.20 ^a^	0.40 ^b^	0.007	0.06 ^ab^	0.02 ^a^	0.11 ^b^	0.036	Small chain store
**Butter, Margarines, mixes**	0.16 ^a^	0.22 ^ab^	0.28 ^b^	0.030	ns	ns	ns	ns	ns
**Other fats**	0.15 ^a^	0.19 ^a^	0.37 ^b^	0.000	ns	ns	ns	ns	ns
**Potatoes**	0.39 ^a^	0.59 ^c^	0.51 ^b^	0.000	ns	ns	ns	ns	ns
**Root vegetables**	0.48 ^a^	0.60 ^b^	0.41 ^a^	0.008	0.06 ^a^	0.04 ^a^	0.15 ^b^	0.038	Small chain store
**Vegetables, except root vegetables**	0.55 ^a^	0.67 ^b^	0.55 ^a^	0.007	ns	ns	ns	ns	ns
**Fresh fruit**	0.50 ^a^	0.69 ^b^	0.54 ^a^	0.002	ns	ns	ns	ns	ns
**Fruit and vegetable preserves (including dried, canned)**	0.28 ^a^	0.35 ^b^	0.45 ^c^	0.045	ns	ns	ns	ns	ns
**Carbonated drinks, still drinks**	0.21 ^a^	0.24 ^a^	0.37 ^b^	0.038	ns	ns	ns	ns	ns
**Coffee, tea, cocoa**	0.15 ^a^	0.14 ^a^	0.32 ^b^	0.000	0.05 ^a^	0.07 ^a^	0.18 ^b^	0.014	Small chain store
**Food concentrates**	0.19 ^a^	0.22 ^a^	0.44 ^b^	0.000	ns	ns	ns	ns	ns
**Ketchup, mayonnaise, mustard and other sauces**	0.23 ^a^	0.29 ^a^	0.48 ^b^	0.000	0.07 ^a^	0.06 ^a^	0.19 ^b^	0.006	Small chain store
**Legume seeds**	0.21 ^a^	0.25 ^a^	0.44 ^b^	0.000	ns	ns	ns	ns	ns
**Preparations from pulses**	0.28	0.27	0.40	ns	ns	ns	ns	ns	ns
**Chilled ready meals**	0.23 ^a^	0.39 ^b^	0.51 ^b^	0.000	ns	ns	ns	ns	ns
**Frozen**	0.23 ^a^	0.34 ^b^	0.41 ^b^	0.004	ns	ns	ns	ns	ns

ns, not significant; an identical letter at the frequency rate means that there are no significant differences between the clusters; * the highest frequency rate means the highest frequency.
